# Acute and chronic effect of bariatric surgery on circulating autotaxin levels

**DOI:** 10.14814/phy2.14004

**Published:** 2019-02-28

**Authors:** Raphaëlle Bourgeois, Marie‐Eve Piché, Audrey Auclair, Thomas Grenier‐Larouche, Patricia L. Mitchell, Paul Poirier, Laurent Biertho, Simon Marceau, Frédéric‐Simon Hould, Simon Biron, Stéfane Lebel, Odette Lescelleur, François Julien, Julie Martin, André Tchernof, Patrick Mathieu, André C. Carpentier, Benoit J. Arsenault

**Affiliations:** ^1^ Centre de recherche de l'Institut universitaire de cardiologie et de pneumologie de Québec Québec Canada; ^2^ Department of Medicine, Faculty of Medicine Université Laval Québec Canada; ^3^ Faculty of Pharmacy Université Laval Québec Canada; ^4^ School of Nutrition Université Laval Québec Canada; ^5^ Department of Surgery, Faculty of Medicine Université Laval Québec Canada; ^6^ Department of Medicine, Division of Endocrinology Centre de recherche du CHUS, Université de Sherbrooke Canada

**Keywords:** Autotaxin, bariatric surgery, caloric restriction, obesity, sex

## Abstract

Autotaxin (ATX), an adipose tissue‐derived lysophospholipase, has been involved in the pathophysiology of cardiometabolic diseases. The impact of bariatric surgery on circulating ATX levels is unknown. We examined the short‐ (24 h, 5 days) and longer‐term (6 and 12 months) impact of bariatric surgery; as well as the short‐term effect of caloric restriction (CR) on plasma ATX levels in patients with severe obesity. We measured ATX levels in 69 men and women (mean age: 41 ± 11 years, body mass index: 49.8 ± 7.1 kg/m^2^), before and after biliopancreatic diversion with duodenal switch surgery (BPD‐DS) as well as in a control group (patients with severe obesity without surgery; *n* = 34). We also measured ATX levels in seven patients with severe obesity and type 2 diabetes who underwent a 3‐day CR protocol before their BPD‐DS. At baseline, ATX levels were positively associated with body mass index, fat mass, insulin resistance (HOMA‐IR) as well as insulin and leptin levels and negatively with fat‐free mass. ATX concentrations decreased 26.2% at 24 h after BPD‐DS (342.9 ± 152.3 pg/mL to 253.2 ± 68.9 pg/mL, *P *< 0.0001) and by 16.4% at 12 months after BPD‐DS (342.9 ± 152.3 pg/mL to 286.8 ± 182.6 pg/mL, *P *= 0.04). ATX concentrations were unchanged during follow‐up in the control group (*P *= 0.4), and not influenced by short‐term CR. In patients with severe obesity, bariatric surgery induced a rapid and sustained decrease in plasma ATX levels. Acute changes in ATX may not be explained by bariatric surgery‐induced CR.

## Introduction

Autotaxin (ATX) is an adipose tissue (AT)‐derived enzyme that may be involved in the pathophysiology of cardiometabolic disease such as atherosclerotic cardiovascular disease (ACVD) and type 2 diabetes (T2D). ATX, which is encoded by the *ENPP2* gene, possesses a lysophospholipase D activity and is involved in the generation of lysophosphatidic acid (lyso‐PA) through the hydrolysis of lysophosphatidyl choline (Morris and Smyth 2016). Lyso‐PA is a bioactive lipid mediator that induces endothelial cell migration, angiogenesis as well as an inflammatory response and lipid accumulation in monocytes (Panetti et al. 2000). Work from Bouchareb et al. (2015) recently showed that lyso‐PA derived from ATX promotes inflammation and the development of calcific aortic valve stenosis (CAVS). Moreover, the ATX‐lyso‐PA axis has been involved in other disorders such as hepatic steatosis (Rachakonda et al. 2015), cancer (Liu et al. 2009; Barbayianni et al. 2015) and atherosclerosis (Smyth et al. 2014).

AT is an active endocrine organ than secretes dozens of adipokines (adipocyte‐derived bioactive cytokines). Although ATX is expressed and secreted by various tissues, the primary site of expression is AT. It has been estimated that approximately 40% of the circulating ATX could be derived from AT (Ferry et al. 2003; Giganti et al. 2008; Dusaulcy et al. 2011). In humans, ATX gene expression, which increases with obesity, might be associated with impaired glucose tolerance and insulin resistance (Boucher et al. 2005). Levels of ATX expression also vary according to fat depots; its expression being higher in subcutaneous fat compared to visceral fat (Rancoule et al. 2012). However, a study found that visceral AT *ENPP2* gene expression was higher in obese individuals compared to non obese whereas subcutaneous AT expression was comparable. The association between circulating ATX levels and obesity is also controversial, with a study showing no association (Rachakonda et al. 2015) and others documenting positive (Reeves et al. 2015) and negative associations (Nishimura et al. 2014).

Although, these findings suggest that the ATX‐lyso‐PA axis could be an important mediator in the association between high‐risk obesity and a broad range of cardiometabolic diseases, whether interventions targeting weight loss influence plasma ATX levels is unknown. Currently, the most effective therapy to induce clinically significant and sustained weight loss is bariatric surgery. Of the various types of commonly performed bariatric surgeries, biliopancreatic diversion with duodenal switch (BPD‐DS) is the most effective for long‐term weight loss and T2D resolution as it induces both caloric restriction (CR) and intestinal malabsorption of nutrients (Buchwald et al. 2009).

The objectives of this study were to investigate the acute and longer‐term effects of BPD‐DS on plasma ATX levels and to examine the impact of a short‐term CR on plasma ATX levels in patients with severe obesity.

## Material and Methods

### Study participants

Patients were recruited at the bariatric surgery clinic of the *Institut universitaire de cardiologie et de pneumologie de Québec* (IUCPQ) as previously described (Boyer et al. 2017). A total of 69 randomly selected men and women with severe obesity (body mass index; BMI ≥ 40 or ≥ 35 kg/m^2^ with associated comorbidities) who were 18 years of age or older were included in this study. Additional exclusion criteria were: use of a pacemaker, hip prosthesis, previous bariatric surgery, age above 65 years, body weight greater than 180 kg, orlistat use 3 months prior to inclusion in the study and psychological condition limiting the odds of a successful follow‐up. The control group included 33 participants with severe obesity selected from the waiting list to age‐ and sex‐matched patients of the BPD‐DS. Blood samples were obtained before BPD‐DS surgery (baseline), and after 24 h, 5 days, 6 and 12 months following surgery. For the control group, blood samples were obtained at baseline, 6 and 12 months. The experimental protocol was approved by the ethics committee of the IUCPQ and all patients gave their written informed consent before being included in the study.

### Anthropometric measurements

Weight, BMI, fat and fat‐free mass were assessed with an electrical bioimpedance balance (Tanita TBF‐310, Tokyo, Japan) following a 12‐h fast. Medical history and pharmacological therapy were collected from clinical file consultations for the presence of T2D, hypertension, cardiovascular disease (CVD), and dyslipidemia.

### Plasma lipid‐lipoprotein profile

After a 12‐h overnight fast, blood samples were collected into Vacutainer tubes containing EDTA for measurement of plasma lipid and lipoprotein levels. Plasma was then aliquoted into microtubes and stored at −80°C until analysis. Glycated hemoglobin (HbA1c) was measured in fresh samples by turbidimetric inhibition immunoassay. Assays were performed in the hospital clinical biochemistry laboratory using standard methodology (fasting plasma glucose, cholesterol, triglyceride and HDL cholesterol) or in the research laboratory. LDL‐cholesterol concentration was calculated using the Friedewald formula. Apolipoprotein B (ApoB) levels were measured by an immunoturbidimetric method (Roche Diagnostics Integra 800 system). Plasma cholesterol, triglyceride, HDL cholesterol and glucose were measured using colorimetric enzymatic kits (cholesterol, triglycerides and HDL cholesterol: Roche Diagnostics Indianapolis, IN, USA; glucose: Wako Chemicals, Richmond, VA, USA). Plasma insulin and adiponectin levels were measured by ELISA (Crystal Chem Inc, Downers Grove, IL, USA). The Homeostatic model assessment of insulin resistance (HOMA‐IR) was calculated from fasting plasma insulin and glucose levels as (insulin x glucose)/22.5, where the insulin concentration is reported as milli‐units per liter and glucose as milli‐molar concentrations. Plasma leptin and ATX levels were measured by ELISA (R&D systems, Minneapolis, MN, catalog number of ATX kit was DENP20).

### Caloric restriction study

Eight patients with T2D who were eligible for BPD‐DS surgery but who did not undergo surgery (3 men and 5 women) were selected for the CR substudy as previously described (Plourde et al. 2014). All participants were weight‐stable for 2 months prior inclusion. Approximately 2 months before surgery, patients underwent a CR protocol during which energy intake matched the average quantity of food ingested routinely before and after BPD‐DS. Patients who undergo bariatric surgery usually receive saline+dextrose infusions to avoid post surgery dehydration. In order to control for the effect of this infusion, we performed saline+dextrose infusions during the CR protocol. We were then able to differentiate the effects of the surgery *per se* and the effect of fasting under saline + dextrose infusion. Study participants were administered a mixed intravenous solution (NaCl 0.9% at 30 mL/h and dextrose 5% at 60 mL/h). Plasma ATX levels were measured in seven of these patients after an overnight fast during a 3‐day CR phase and before and after BPD‐DS surgery. In these participants, the CR protocol and BPD‐DS surgery were performed approximately 2 months apart.

### Statistical analyses

Student *t*‐tests and chi‐square tests were respectively used to assess differences between continuous and categorical clinical variables at baseline in the surgery vs. control group. Student *t*‐tests were also used to test the differences in plasma ATX levels across patient subcategories (sex and T2D status). For the bariatric surgery patients, to analyze significant changes among scheduled endpoints measurements (baseline, 24‐h, 5 days, 6 and 12 months), data were analyzed using a mixed model with two experimental factors defined: (1) one linked to the variability among patients, a random factor and (2) the other associated to the comparison among the different time periods, a fixed factor. The latter was analyzed as a repeated‐measure factor with the use of a heterogeneous compound symmetric covariance structure. To compare with the control group at baseline, 6 and 12 months, a second fixed factor was introduced in the statistical mixed model with an interaction term. The impact of CR vs. bariatric surgery was also tested using the statistical mixed model with an interaction term. Spearman rank correlation coefficients were computed to determine the association between ATX levels and other cardiometabolic parameters at baseline. All statistical analyses were performed with SAS (v9.3, Cary, NC, USA).

## Results

The study sample included 69 patients undergoing BPD‐DS surgery and 33 controls with severe obesity. The anthropometric and clinical characteristics of patients at baseline are presented in Table 1. Patients undergoing bariatric surgery had a higher BMI compared to controls (49.9 ± 7.0 vs. 42.2 ± 10.3 kg/m^2^; *P* = 0.005) and higher fat mass in the surgery group (70.6 ± 17.5 kg surgery group vs. 59.9 ± 17.8 kg controls; *P* = 0.006). Baseline levels of adiponectin were lower and leptin levels were higher in the surgery group compared to controls. No difference was observed between the surgery and control groups with regards to baseline levels of ATX.

**Table 1:** Baseline characteristics of study participants

	Bariatric surgery	Controls	*P*‐value
Number of participants	69	33	
Age, years	41.5 (11.1)	42.2 (10.3)	0.7
Men, *n* (%)	20 (29.0)	11 (32.4)	0.7
Statin users, *n* (%)	23 (33.3)	17 (54.8)	<0.0001
Smokers, *n* (%)	13 (18.8)	8 (25.8)	<0.0001
Type 2 diabetes, *n* (%)	34 (49.3)	10 (32.3)	<0.0001
Body weight, kg	136.6 (26.4)	127.1 (28.4)	0.1
Body mass index, kg/m^2^	49.9 (7.0)	45.3 (7.6)	0.005
Fat percent, %	51.4 (5.5)	47.4 (6.2)	0.002
Fat mass, kg	70.6 (17.5)	59.9 (17.8)	0.006
Fat‐free mass, kg	66.0 (13.5)	66.1 (15.9)	0.9
LDL cholesterol, mmol/L	2.61 (0.72)	2.56 (0.87)	0.7
HDL cholesterol, mmol/L	1.27 (0.29)	1.16 (0.30)	0.1
Triglycerides, mmol/L	1.70 (1.07)	1.83 (0.75)	0.7
Apolipoprotein B, g/L	0.78 (0.19)	0.80 (0.20)	0.6
Insulin, pmol/L	199.4 (133.5)	175.5 (136.6)	0.4
Glucose, mmol/L	6.77 (2.40)	6.62 (2.91)	0.9
HOMA‐IR	8.92 (7.15)	7.79 (6.44)	0.4
HbA1c, %	6.24 (1.05)	6.45 (1.30)	0.3
Adiponectin, ng/mL	5414 (3531)	8969 (8382)	0.04
Leptin, pg/mL	69,354 (46,383)	43,559 (31,397)	0.003
Autotaxin, ng/mL	342.0 (156.1)	303.3 (146.0)	0.2

Data are presented as mean (±SD). LDL: low‐density lipoprotein; HDL: high‐density lipoprotein; HOMA‐IR: homeostatic model of insulin resistance; HbA1c: glycated hemoglobin.

**Figure 1:**
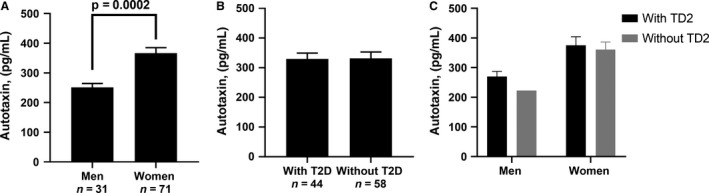
Baseline autotaxin levels according to sex (A), type 2 diabetes (T2D) status (B) and sex and type 2 diabetes status combined (C).

Investigating the potential differences in ATX levels at baseline between patient subgroups, we found differences according to sex (Fig. 1A, 244.7 ± 67.8 in men vs. 366.5 ± 164.1 pg/mL in women; *P* = 0.0002) but not according to T2D status (Fig. 1B), even when considering men and women separately (Fig. 1C).

At baseline, ATX levels were positively associated with BMI, fat mass, insulin, insulin resistance (HOMA‐IR), and leptin and negatively with fat‐free mass (Table 2). These correlations were also observed when considering men or women separately for BMI, insulin and HOMA‐IR. We also observed a positive correlation with fat‐free mass and glucose in women only. At 6 months (Table 3), ATX levels were correlated positively with fat percent, triglycerides, insulin, HbA1c, and HOMA‐IR, and negatively with fat‐free mass. None of these correlations remained significant when considering men only, but most of them (triglycerides, insulin, HbA1c, HoMA‐IR) were still significantly correlated in women. Glucose levels were also positively associated with ATX levels in women. At 12 months (Table 4), ATX levels were still associated with fat percent, fat‐free mass, and also associated with apoB and leptin. None of these associations remained significant in both men and women. Triglycerides remained positively associated with ATX levels in women.

**Table 2:** Association between autotaxin and cardiometabolic risk factors at baseline

	All		Men		Women	
Number of participants	69		20		49	
	*r*	*P*	*r*	*P*	*r*	*P*
Age	‐0.11	0.26	‐0.10	0.59	0.03	0.80
Body weight	‐0.08	0.43	0.34	0.06	0.21	0.08
BMI	0.23	**0.02**	0.46	**0.01**	0.35	**0.003**
Fat percent	0.27	**0.01**	0.34	0.06	0.05	0.68
Fat mass	0.12	0.24	0.38	**0.03**	0.18	0.14
Fat‐free mass	‐0.22	**0.03**	0.03	0.87	0.25	**0.04**
Apo B	0.03	0.74	‐0.05	0.78	0.06	0.64
LDL‐C	0.03	0.77	‐0.11	0.56	0.02	0.85
HDL‐C	0.10	0.30	‐0.06	0.76	‐0.02	0.86
Triglycerides	‐0.02	0.81	‐0.18	0.32	0.18	0.13
Insulin	0.24	**0.01**	0.67	**<.0001**	0.35	**0.003**
Glucose	0.18	0.08	0.11	0.55	0.31	**0.01**
HbA1c	0.03	0.76	0.26	0.15	0.17	0.16
HOMA‐IR	0.27	**0.01**	0.59	**0.0004**	0.37	**0.002**
Adiponectin	0.04	0.71	0.18	0.36	‐0.01	0.95
Leptin	0.38	**0.0002**	0.52	**0.01**	0.06	0.62

LDL: low‐density lipoprotein, HDL: high‐density lipoprotein, HOMA‐IR: homeostatic model of insulin resistance, HbA1c: glycated hemoglobin.

*P*‐values indicate statistically significant correlations (*P* < 0.05) in bold.

**Table 3:** Association between autotaxin and cardiometabolic risk factors at 6 months

	All		Men		Women	
Number of participants	69		20		49	
	*r*	*P*	*r*	*P*	*r*	*P*
Age	−0.10	0.42	−0.04	0.86	0.01	0.95
Body weight	−0.12	0.33	0.37	0.12	0.13	0.38
BMI	0.17	0.17	0.19	0.44	0.19	0.21
Fat percent	0.40	**0.0009**	0.21	0.38	0.03	0.85
Fat mass	0.20	0.12	0.26	0.29	0.09	0.56
Fat‐free mass	−0.31	**0.01**	0.18	0.47	0.21	0.16
Apo B	0.17	0.18	−0.09	0.71	0.25	0.09
LDL‐C	0.10	0.43	0.18	0.46	0.05	0.74
HDL‐C	0.10	0.47	0.003	0.99	0.22	0.13
Triglycerides	0.26	**0.04**	−0.31	0.20	0.32	**0.03**
Insulin	0.31	**0.01**	0.22	0.36	0.48	**0.0006**
Glucose	0.14	0.28	0.006	0.98	0.32	**0.03**
HbA1c	0.31	**0.02**	−0.22	0.39	0.42	**0.009**
HOMA‐IR	0.30	**0.01**	0.20	0.41	0.48	**0.0007**
Adiponectin	−0.02	0.89	0.22	0.37	−0.12	0.44
Leptin	0.24	0.06	0.14	0.56	−0.17	0.27

LDL: low‐density lipoprotein; HDL: high‐density lipoprotein; HOMA‐IR: homeostatic model of insulin resistance; HbA1c: glycated hemoglobin.

*P*‐values indicate statistically significant correlations (*P* < 0.05) in bold.

**Table 4:** Association between autotaxin and cardiometabolic risk factors at 12 months

	All		Men		Women	
Number of participants	69		20		49	
	*r*	*P*	*r*	*P*	*r*	*P*
Age	−0.01	0.93	0.13	0.58	0.09	0.53
Body weight	−0.15	0.22	0.39	0.09	0.05	0.73
BMI	0.09	0.46	0.37	0.11	0.07	0.65
Fat percent	0.28	**0.02**	0.30	0.20	−0.10	0.50
Fat mass	0.15	0.21	0.29	0.21	−0.03	0.82
Fat‐free mass	−0.30	**0.01**	0.21	0.39	0.14	0.33
Apo B	0.25	**0.04**	0.20	0.39	0.23	0.12
LDL‐C	0.22	0.06	0.28	0.23	0.12	0.41
HDL‐C	0.09	0.46	−0.25	0.29	−0.02	0.88
Triglycerides	0.20	0.09	−0.08	0.73	0.31	**0.03**
Insulin	0.11	0.37	0.12	0.62	0.21	0.14
Glucose	0.006	0.96	0.36	0.12	0.08	0.60
HbA1c	0.03	0.82	0.07	0.77	0.09	0.56
HOMA‐IR	0.10	0.41	0.16	0.49	0.21	0.16
Adiponectin	0.07	0.56	−0.04	0.88	0.06	0.66
Leptin	0.27	**0.03**	0.08	0.74	0.09	0.52

LDL: low‐density lipoprotein; HDL: high‐density lipoprotein; HOMA‐IR: homeostatic model of insulin resistance; HbA1c: glycated hemoglobin.

*P*‐values indicate statistically significant correlations (*P* < 0.05) in bold.

**Figure 2:**
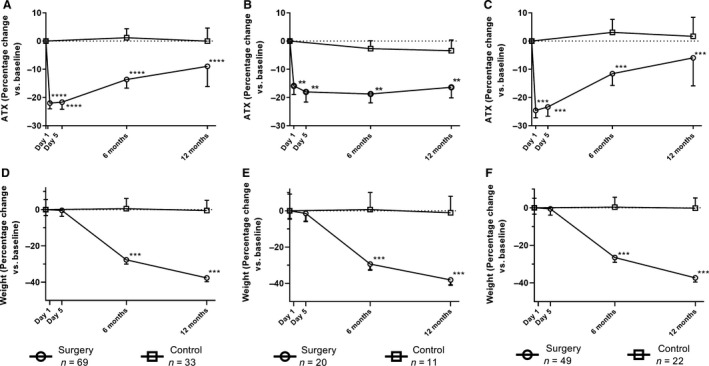
Acute and longer‐term changes in ATX concentrations in patients who underwent bariatric surgery and in controls (A) and separately in men (B) and in women (C) and in weight in patients who underwent bariatric surgery and in controls (D) and separately in men (E) and in women (F). **P ≤ 0.01; ***P ≤ 0.001; ****P ≤ 0.0001.

Changes in ATX levels and body weight during follow‐up are shown for both control and surgery groups in Figure 2. ATX levels decreased significantly 24 h after surgery and remained significantly lower at 5 days, 6 and 12 months post‐surgery compared to baseline levels. Significant time‐group interactions were observed (*P* = 0.002). Because we found a sex difference in ATX levels, we assessed changes in men (Fig. 2B) and women (Fig. 2C) separately. Although ATX levels were significantly influenced by bariatric surgery in both sexes, the impact appeared to be slightly more sustained in men.

In the bariatric surgery group, 6‐month changes in ATX levels correlated with 6‐month changes in triglyceride levels (*r* = 0.27, *P* = 0.03) while 6‐month changes in ATX levels correlated with 6‐month changes in insulin levels in men only (*r* = 0.67, *P* = 0.002). In the bariatric surgery group, 12‐month changes in ATX levels correlated with 12‐month changes in triglyceride levels (*r* = 0.27, *P* = 0.03) and with 12‐month changes in insulin levels in men and women (*r* = 0.31, *P* = 0.01).

**Figure 3:**
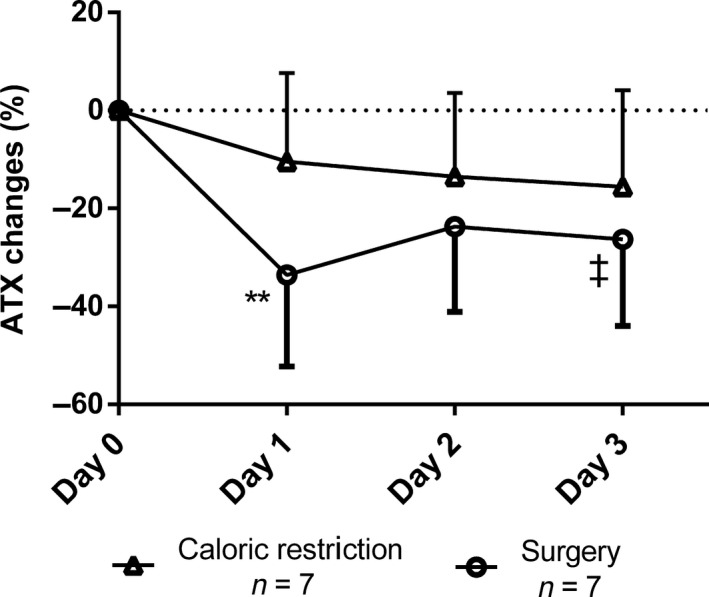
Changes in autotaxin concentrations in patients who underwent caloric restriction before the bariatric surgery procedure. ‡P ≤ 0.1; **P ≤ 0.01.

To determine if acute changes in ATX levels after bariatric surgery were related to the CR induced by BPD‐DS surgery, seven participants with severe obesity and T2D were submitted to a 3‐day CR protocol prior to their bariatric surgery that matched the energy intake that we generally observe during the 3 days after BPD‐DS surgery. Results presented in Figure 3 show that although ATX levels appeared to be lower during the CR protocol, changes in ATX were not statistically significant. However, when the same study participants underwent BPD‐DS surgery (on average 2 months later), comparable results (Fig. 2), that is a reduction in ATX levels (−33.5% ± 50.1% 24 h after surgery) were obtained.

## Discussion

ATX has been shown to be involved in obesity and impaired glucose metabolism, in both human (Reeves et al. 2015) and animal models (Rancoule et al. 2012), but the impact of severe obesity and the effect of massive AT loss on ATX levels has not been investigated. In this study, we show that severely obese women have higher ATX levels than men and that higher body weight is positively correlated with ATX levels in both men and women. Our results also suggest that bariatric surgery induces a rapid and sustained decrease in ATX levels over time, and that the short‐term effect of bariatric surgery on ATX levels may not be explained by bariatric surgery‐associated CR.

It is well known that bariatric surgery induces a clinically significant weight loss. Studies investigating the potential association between BMI and ATX levels have yielded conflicting results. The study by Nsaibia et al. (2016) found a small but positive correlation between BMI and ATX mass, but not with ATX enzymatic activity, whereas Reeves et al. (2015) did not find any correlation, except when adjusting for sex. Moreover, Reeves et al. (2015) did not find any correlation between BMI and ATX expression in visceral and subcutaneous fat. Similar to Reeves et al. (2015), we found sex differences in ATX levels. A possible explanation is the level of ATX expression is higher in subcutaneous compared to visceral AT (Giganti et al. 2008) and women tend to accumulate more subcutaneous fat than men (Lemieux et al. 1993). Alternatively, there may be a link between circulating ATX and sex hormones. It was shown that testosterone/estrone ratios were lower in female than in males, regardless of age, and that some adipokines as leptin are higher in female (Cicero et al. 2011). Androgens and estrogens also influence adipogenesis and AT metabolism (Mattsson and Olsson 2007; Tchernof et al. 2015).

Even if AT‐ATX expression is significantly higher in patients exhibiting insulin resistance and impaired glucose tolerance, we did not observe any difference in plasma ATX levels between patients with versus without T2D patients. Nevertheless, all patients with T2D were receiving treatment, thus their glucose levels were controlled. We also observed a rapid and significant decrease in ATX levels immediately post surgery (measured at 24 h and 5 days) prior to any loss of fat mass (136.6 ± 26.4 kg at baseline vs. 135.9 ± 26.5 kg at 5 days post surgery). Several factors may explain this finding. First, studies have suggested that plasma glucose and insulin levels may influence ATX level (Boucher et al. 2005; Rancoule et al. 2012) *in vitro*. D'Souza et al. (2017) showed that ATX was regulated by glucose and insulin in adipocytes and that incubation with insulin lead to a decrease in ATX levels after 24 h. Given the almost immediate improvement in insulin sensitivity appearing within days (Guidone et al. 2006; Mari et al. 2006), the decrease in ATX levels could be explained by rapid improvements in insulin sensitivity following bariatric surgery. However, as previously shown in this cohort, CR improves insulin sensitivity to the same extent and as rapidly as BPD‐DS and did not significantly reduce ATX levels in the present study (Plourde et al. 2014). Another hypothesis involves the role of intestinal enteroendocrine cells and their suggested role in the secretion of ATX. In a recent study, Bolier et al. (2016) reported high expression of ATX‐mRNA in the small intestine and identified extensive ATX expression in human enteroendocrine cells. According to that study, an intervention leading to a bypass of a small or large part of the small intestine could then lead to a reduction in ATX. Because BPD‐DS involves a decrease in stomach volume through a sleeve gastrectomy and bypasses most of the jejunum, the number of functional enteroendocrine cells in contact with nutrients is reduced, possibly leading to a decrease of ATX secretion. More studies on the role of these cells in the regulation of ATX levels are needed. The bariatric surgery procedure could also influence ATX activity through bile acids, which can inhibit enzymatic ATX activity (Keune et al. 2016). Indeed, some studies showed that individuals with excess body weight have lower bile acid concentrations, and bariatric procedures can induce an increase in bile acid concentrations after 3 and 12 months (Steinert et al. 2013). These effects are, however, modulated by fasting/feeding status as well as time since surgery (Dutia et al. 2016). The third reason for this rapid decrease could have involved surgery‐induced CR. Given the physical restriction of the stomach size, patients consume smaller meal portions and are submitted to CR. We did not observe a decrease in ATX levels with CR, which does not support this third hypothesis.

Another possible explanation for the rapid and sustained decrease in ATX concentrations following bariatric surgery could be attributable to the change in inflammatory status. It is known (D'Souza et al. 2018) that ATX secretion by adipocytes could be increased by inflammatory cytokines such as TNF*α* or IL6 (Kelly et al. 2015). Also, in a previous study, bariatric surgery leads to an improvement of inflammatory markers or adipokines. However, in our study, changes in TNF*α* and IL6 were not correlated with the changes in ATX levels (data not shown). Finally, after various weight‐loss approaches, subcutaneous fat loss is greater than visceral fat loss on an absolute basis (Merlotti et al. 2017). Because ATX is more highly expressed in subcutaneous AT, mobilization of larger amounts of this compartment could have contributed to the decrease ATX levels. Yet, fat loss was minimal early after the BPD‐DS.

Our study has limitation. First, it must be acknowledged that the CR study is limited by a small size (*n* = 7 patients) and that we may have lacked power to detect smaller associations. This sample however was large enough to document changes in plasma PCSK9 levels with CR (Keune et al. 2016). Second, our study documents the impact of DBP‐DS on ATX levels and our results may not be attributable to other types of bariatric surgeries that may be associated with lower levels of weight loss. Third, we used an assay that measures total ATX levels and not specifically ATX that might be bound to plasma proteins such as albumin or lipoproteins. This assay also does not provide information on ATX activity.

In conclusion, our study is the first to investigate the effect of weight loss induced by bariatric surgery on circulating levels of ATX. We found that in patients with severe obesity, bariatric surgery led to a rapid and sustained decrease in plasma ATX levels. Acute changes in ATX however, may not be explained by bariatric surgery‐induced CR. These results increase our understanding of the factors that influence ATX metabolism in humans.
